# Supplementary material for: Monkeypox Post-COVID-19: Knowledge, Worrying, and Vaccine Adoption in the Arabic General Population
Source: Vaccines (Basel). 2023 Mar 29;11(4):759. doi: 10.3390/vaccines11040759 (PMC10141174; doi:10.3390/vaccines11040759)
Supplement: Supplementary file 1 [file vaccines-11-00759-s001.zip › vaccines-2212947-supplementary.pdf]

Supplementary Materials

THE QUESTIONNAIRE

Consent to Participating

Do you agree to participate in this survey?

- Yes

- No

Sociodemographic Variables

Country:

……………………

Age (in years):

……………………

Gender:

- Male

- Female

Marital status:

- Single

- Married

- Widower

- Divorced

Place of residency:

City/ countryside

Economic status:

- Bad
- Moderate
- Good
- Excellent

Chronic diseases (hypertension, diabetes…):

- Yes
- No

Attitudes, Perceptions, and Beliefs about Monkeypox

Have you ever had COVID-19?

- Yes
- No

Did you and your family adhere to the precautionary measures during the COVID-19 pandemic?

- Little adherence
- Medium adherence
- Always adherence

How worried are you about monkeypox compared to COVID-19?

- I am more concerned about COVID-19
- I am more concerned about monkeypox

The main reasons you may be concerned about monkeypox:

- Worry about yourself and your family getting monkeypox
- Worried about another global pandemic
- Worried about a global quarantine due to monkeypox
- Concerned about the occurrence of suspension of international flights
- Other concerns

Do you consider monkeypox to be a serious and virulent disease that calls for respiratory precautions and precautions around contact with others?

- Yes
- No

What are your concerns about monkeypox (multiply answer is available):

- Worry about closing airports and flights
- Worried about a quarantine
- Worry about yourself and your family
- Worry about a global pandemic
- Other concerns

Monkeypox Disease Knowledge

Please answer each question with (yes/ no/ do not know)

- There are many cases of monkeypox infection among humans in the Arab world
- Monkeypox is common in Southeast Asian countries
- There is an epidemic of human monkeypox around the world
- Monkeypox is a viral disease
- Monkeypox is easily transmitted from person to person

- Monkeypox and smallpox have similar signs and symptoms
- A skin rash is a sign or symptom of human monkeypox
- Papules on the skin are a sign or symptom of human monkeypox
- Antibiotics are required in the management of monkeypox in humans
- Diarrhea is one of the signs or symptoms of monkeypox in humans
- Vaccination is available to prevent human monkeypox

Acceptance to Receive Vaccination Against Monkeypox Disease

Do you accept receiving monkeypox vaccine?

- Yes
- No

In your opinion, who are the most likely candidates to receive the monkeypox vaccine? (Multiply answer is available):

- Immunocompromised (have weakened immunity)
- Elderly
- Children
- People with chronic diseases
- Workers in the health sector
- Cancer patients
- Teachers
- Other cases

GAD7 Scale's Items

Over the past two weeks, how often have the following problems bothered you?

(Please answer each question with never at all/ several days/ more than half of the days/ almost every day)

- Feeling stressed or anxious about monkeypox
- Inability to stop or control your anxiety about monkeypox
- Worrying a lot about monkeypox
- You have difficulty relaxing when you think of monkeypox
- Being unable to feel relief or inner peace at the thought of monkeypox
- You get upset easily when you hear of monkeypox
- You feel scared, as if something terrible might happen upon hearing of monkeypox
